# Incidence trend of type 1 diabetes mellitus in Serbia

**DOI:** 10.1186/s12902-020-0504-y

**Published:** 2020-03-09

**Authors:** Ciric Vojislav, Rancic Natasa, Pesic Milica, Antic Slobodan, Kocic Radivoj, Radojkovic Danijela, Radenkovic Sasa

**Affiliations:** 1 University of Niš, Faculty of Medicine Niš, Niš, Serbia; 2 Clinic for Endocrinology, Diabetes and Metabolic Diseases, Clinical Center Niš, Niš, Serbia; 3 Institute for Public Health Niš, Niš, Serbia

**Keywords:** Type 1 diabetes mellitus, Incidence, Trend, Age differences

## Abstract

**Background:**

The incidence of type 1 diabetes mellitus (T1DM) increased worldwide.

The objective of the paper was to compare the incidence trend of T1DM in children and adolescents aged 0–19 and in adults under 30 years of age in Serbia from 2006 to 2017. Additional aim was to compare incidence rates of T1DM and type 2 diabetes mellitus (T2DM) among adults aged 20–24 and 25–29 years of age.

**Methods:**

Trends and annual percentage change (APC) of the incidence rate with corresponding 95% confidence intervals (CI) were calculated by Joinpoint Regression Analyses.

**Results:**

We found a significant increase of incidence in children aged 5–9 with the APC of 5.7% (95%CI: 2.3–9.1), and in children aged 10–14 with the APC of 2.1% (95%CI: 0.6–3.6). A significant decrease of incidence was determined in adolescents aged 15–19 with the APC -4.9% (95%CI: − 8.9 to – 0.7) and in adults aged 25–29 with the APC -7.3% (95%CI: − 12.5 to − 1.8).

**Conclusion:**

The increase of incidence in children aged 0–14 and its decrease after 15 years of age showed that T1DM is predominantly a metabolic disease of children in Serbia. A significant increase in incidence was recorded in two age groups, namely 5–9 and 10–14 years of age. The highest increase was in children aged 5–9 and the highest incidence rate was in children aged 10–14. An insignificant increasing of T2DM incidence was observed in young adults aged 25–29. The increase in incidence rates in children, but not in young adults, suggests that the precipitating factors of children-onset disease may differ from those of adult-onset T1DM.

## Background

Type 1 diabetes mellitus (T1DM) can develop at any age but it is the most common metabolic disease in children and youth with incidence increased by 2–5% worldwide (5.3% in North America, 4.0% in Asia and 3.2% in Europe) with the exception of South America and the West India where T1DM is less prevalent and where there is a 3.6% decrease in incidence [1, 2].

The highest incidence (> 20/100,000 per year) was reported in Sardinia, Finland, Sweden, Norway, Portugal, the United Kingdom (UK), Canada and New Zealand. The lowest incidence (< 1/100,000 per year) was reported in the populations from China and South America. In Africa, the incidence is intermediate and in Asia it is low, with the recorded exception of Kuwait [3–5].

T1DM contributes to 5–10% of the total cases of diabetes mainly in children and youth worldwide [6] and in Serbia T1DM accounts for 5% of all registered DM patients [7].

T1DM is a heterogeneous disease characterized by the autoimmune destruction of the beta pancreatic cells that produce insulin, which leads to severe insulin deficiency and which is followed by the raising of blood glucose levels [8]. The most common causes of T1DM subtype (Type 1a) are both genetic predisposition and autoantibodies. A small number of T1DM cases results from an idiopathic destruction or failure of beta pancreatic cells (Type 1b), [9–11].

Interaction between environmental factors triggers autoimmune response in genetically susceptible individuals [12] and the majority of the international variations in the incidence has been attributed to exposure to risk factors related to environment. The environmental factors, such as exposure to known or suspected risk factors related to nutrition (cow’s milk, breastfeeding, wheat gluten, and vitamins D and E) [12] and exposure to a number of toxic chemicals (bisphenol, dioxins, persistent organic pollutants, pesticides), chemical wastes and exposure to some viral infections have also been linked to the risk of developing T1DM [13].

Prevalence of childhood obesity has significantly increased from < 1% in 1975 to 7.8 and 5.6% in 2016 for boys and girls worldwide [14]. According to the new findings, children with T1DM were heavier than their peers before clinical manifestation of the disease and there is overall evidence supporting the association between childhood obesity or higher Body Mass Index (BMI) and subsequent greater risk for the development of T1DM [14, 15].

It is considered that overweight and obesity are now prevalent among children and young adults with T1DM, and the established “accelerator hypothesis” explained the association between that greater body weight and the increased demand for peripheral insulin. Appearance of glucotoxicity and lipotoxicity which is determined as the ectopic lipid disposition in pancreatic islets can act as additional accelerators of beta cell apoptosis and the subsequent onset of T1DM [14, 15].

Data of T1DM incidence in Sweden has been partially attributed to the good control of childhood obesity [16].

The greatest number of new T1DM cases are typically diagnosed in childhood and the peak age is 10–14 [16, 17]. About 10% of adults first diagnosed as type 2 diabetes mellitus (T2DM) were found to have antibodies to beta pancreatic cells. Onset of T1DM 1 in adolescents and young adults older than 15 years of age might have a longer asymptomatic period before clinical presentation of the disease because of the lesser destruction of pancreatic beta cells, less severe loss of insulin secretion and lower frequencies of multiple antibodies than in children with this DM type [18].

While boys and girls are almost equally affected with T1DM, in adulthood males have 50–70% higher risk to suffer from this type of DM than females [19]. The age at which T1DM starts might be an important factor for excess risk of premature death and higher cardiovascular risk in these patients [20]. Females who were diagnosed with T1DM before the age of 10 are exposed to a higher risk of developing cardiovascular complications and significant diminishing of life expectancy [21].

Because there is no sufficient data concerning the T1DM incidence in young adults compared to data concerning children in Serbia we set up these objectives in the paper.

The first objective of the paper was to determine and to compare the incidence trend of T1DM in children and youth aged 0–19 and in adults under 30 years of age. The additional objective was to compare incidence rates of T1DM and T2DM among adults aged 20–24 and 25–29.

## Methods

A descriptive study was performed. The data about the T1DM incidence were obtained from the Population-Based Serbian Diabetes Registry for the period 2006–2017 [7]. Data were analyzed only for the population of Serbia, because data from the territory of the Autonomous Province of Kosovo and Metohija have been unavailable since 1998. The Register was established in 1980. Now days new set up of the Serbian Diabetes Registry implied its decentralization. The regional Registries were established and they are kept on the level of the administrative districts and are located at the Institutes of Public Health. The database for the entire Serbia is managed by the “Dr Milan Jovanovic Batut” Institute of Public Health of Serbia [7]. The notification of new cases and the deceased from DM is obligatory by law in Serbia. Diagnostic criteria are presented in Table 1.

### Statistical analysis

All the calculations were done by SPSS software package version 18.0 and S-PLAS program, version 2000.

Three types of incidence rates were calculated: crude, age-specific, and age-standardized, calculated by the direct method using the Segi’s World Population as the Standard [22] and expressed as new cases per 100.000 persons. The population data was obtained from the 2002 and 2011 Censuses. Age-specific rates were computed for 5-year age groups; 0–4,5-9,10–14,15–19, 20–24 and 25–29.

In order to compare average annual rates of T1DM incidence among different age groups a Chi-squared test was calculated. Trends and annual percentage change (APC) of the incidence rate with corresponding 95% confidence intervals (CI) were calculated by Joinpoint Regression Analyses. For the regression analyses, the Joinpoint Regression Program version 4.4.0.0. was used (available at http: //surveillance.cancer.gov/joinpoint) [23]. Age-Standardized Rates (ASR) were dependent variable and year was the independent variable. The trend was considered to be significantly increasing (positive change) or decreasing (negative change) when the *p*-value was below 0.05 (*p* < 0.05).

## Results

In the period from 2006 to 2017 the total number of new diagnostic cases of T1DM in individuals from 0 up to 30 years of age was 3506 (1916 males and 1590 females). Newly diagnosed persons aged 0–14 years of age accounted for 58.1%, adolescents aged 15–19 years of age represented 16.5 and 25.4% of all new cases of T1DM were registered in young adults aged 20–29 years of age. In the age group 0–14 years of age boys accounted for 51.7% and girls 48.3%. There were 1045 new cases of T2DM in young adults aged 20–29.

During the observed period, the highest annual Age-Standardized Rate (ASR) of incidence was in the age group of 10–14 and it ranged from 18.84/100,000 (2006) to 25.58/100,000 in 2016, (in average 21/100,000), and the lowest was in the age group of 25–29, ranging from 3.09/100,000 (2015) to 14.20/100,000 (2011), (in average 8.22/100, 000). The twice as lower average annual incidence rate was registered in the age group 15–19 in comparison to the 10–14 age group (Table 2). Annual incidence rate was lower in adults aged 20–24 and 25–29 years of age compared with all age groups in children from 0 to 14 years of age (Table 2).

**Table 1:** Diagnostic criteria for diabetes and related stages of impaired glucose homeostasis [2]

Based on subsequent values of glycemia	Based on the value of glycemia during an OGTT:			
(2 values of glycemia in 2 subsequent days):				
Normal fasting plasma glucose concentration	Normal glucose tolerance			
Fasting plasma glucose concentration < 6,1 mmol/L	Plasma glucose concentration during an OGTT in the			
(< 110 mg/dL)	120th	minute < 7,8 mmol/L (< 140 mg/dL)		
Impaired Fasting Glycaemia (IFG)	Impaired Glucose Tolerance (IGT)			
Fasting plasma glucose concentration ≥ 6,1 mmol/L	Plasma glucose concentration during an OGTT in the			
(110 mg/dL) and < 7,0 mmol/L (126 mg/dL)	120th	minute between 7,8 mmol/L (140 mg/dL) and		
		11,1 mmol/L (200 mg/dL)		
Diabetes Mellitus	Diabetes Mellitus			
Fasting plasma glucose concentration ≥ 7,0 mmol/L	Plasma glucose concentration during an OGTT in the			
(126 mg/dL) or glycemia in any random blood sample	120th	minute ≥11,1 mmol/L (200 mg/dL)		
(regardless of meals) ≥ 11,1 mmol/L (200 mg/dL)				
with the presence of typical diabetes symptoms				
(polyuria, polydipsia, weight loss)

**Table 2:** Annual Age-Standardized Rates of T1DM incidence in Serbia from 2006 to 2017 by age

Annual Age-Standardized Rate of Incidence							
	^a^Number of cases	0–4	5–9	10–14	15–19	20–24	25–29
2006	361	8.43	11.52	18.84	14.45	14.61	13.37
2007	302	3.49	16.15	19.49	13.89	8.19	9.35
2008	274	9.68	9.18	19.96	8.04	5.28	8.79
2009	290	6.24	16.67	21.07	9.59	8.32	8.40
2010	309	9.52	15.42	19.03	15.60	8.12	8.24
2011	326	12.01	16.58	20.64	10.51	9.63	14.20
2012	298	9.34	18.53	23.65	9.75	8.90	5.82
2013	260	8.45	19.70	19.91	8.24	7.46	6.75
2014	281	11.52	19.69	19.76	10.39	9.26	6.90
2015	221	7.30	19.63	20.74	5.80	5.05	3.09
2016	291	9.45	24.04	25.58	8.21	7.10	5.61
2017	293	7.96	18.39	25.38	8.56	7.52	6.86
average annual Standardized Rate		8.56	17.00	21.10	10.42	8.36	8.22

^a^Number of cases in age group 0–29

**Table 3:** Changes of T1DM incidence rates in age groups from 0 to 29 years of age in Serbia in the period from 2006 to 2017: results of Joinpoint Analysis

Group	Lower	Upper	^a^APC	95% CI Lower CI	Upper CI	*p*
0–4	2006	2017	3.1	−2.9	9.5	0.278
5–9	2006	2017	5.7	2.3	9.1	**0.004**
10–14	2006	2017	2.1	0.6	3.6	**0.011**
15–19	2006	2017	−4.9	−8.9	−0.7	**0.026**
20–24	2006	2017	−3	−7.7	1.9	0.201
25–29	2006	2017	−7.3	−12.5	−1.8	**0.015**

^a^Average Percentage Change-APC

**Table 4:** The comparison of the average annual age-standardized incidence rates among age groups by Chi-squared test

Group 1 T1DM	Group2 T1DM	^a^ASR 1	^a^ASR 2	Chi^1^	*p*
0–4	5–9	8.56	17.00	9.66	**0.002**
0–4	10–14	8.56	21.10	18.59	**0.000**
0–4	15–19	8.56	10.42	0.67	0.414
0–4	20–24	8.56	8.36	0.01	0.922
0–4	25–29	8.56	8.22	0.03	0.866
5–9	10–14	17.00	21.10	1.60	0.207
5–9	15–19	17.00	10.42	6.04	**0.014**
5–9	20–24	17.00	8.36	12.17	**0.000**
5–9	25–29	17.00	8.22	13.21	**0.000**
10–14	15–19	21.10	10.42	14.16	**0.000**
10–14	20–24	21.10	8.36	23.38	**0.000**
10–14	25–29	21.10	8.22	25.20	**0.000**
15–19	20–24	10.42	8.36	0.96	0.326
15–19	25–29	10.42	8.22	1.15	0.283
20–24	25–29	8.36	8.22	0.01	0.941

^a^average annual Age-Standardized Rate-ASR

1 Chi-squared test

**Table 5:** The comparison of the average annual age-standardized incidence rates among young adults aged 20–24 and 25–29 years of age by type of DM

Age-group and DM type	Age-group and DM type	^a^ASR1	^a^ASR2	Chi ^1^	*p*
20–24 T1DM	20–24 T2DM	8.36	8.45	0.81	0.368
25–29 T1DM	25–29 T2DM	8.22	11.75	3.70	0.054

^a^average annual Age-Standardized Rate-ASR

1 Chi-squared test

**Fig. 1:**
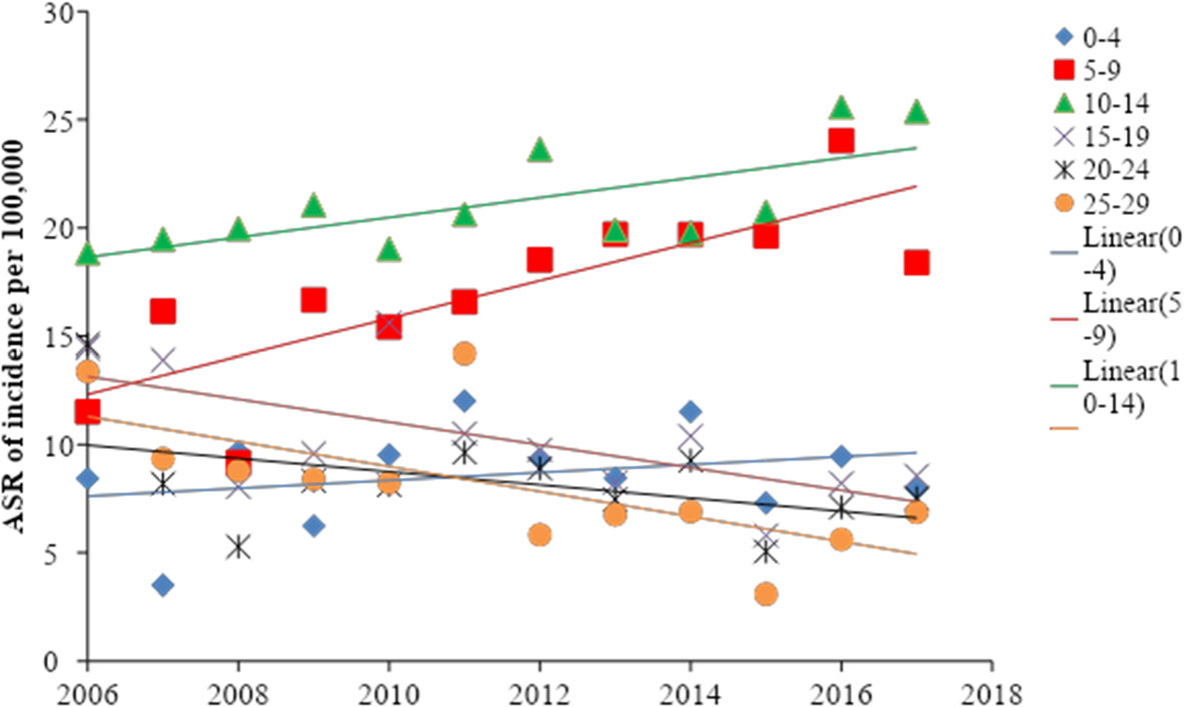
Incidence trend of diabetes mellitus type 1 based on the age-standardized incidence rates in Serbia from 2006 to 2017

Figure 1 and Table 3 show the results of Joinpoint regression analysis of T1DM age-standardized incidence rates in Serbia, during the period 2006–2017.

Figure 1 presents the results of the Jointpoint regression analysis of the incidence trends based on the age-standardized incidence rates of T1DM in Serbia in the period from 2006 to 2017.

During the 12-years observation period there was a significant increase of incidence in children from 5 to 9 age group with the APC of 5.7% yearly (95%CI: 2.3–9.1), whereas the age group 10–14 had APC of 2.1% yearly (95%CI: 0.6–3.6). A significant decreasing trend of incidence was determined in adolescents aged 15–19 with the APC of − 4.9% yearly (95%CI: − 8.9 to – 0.7) and in adults 25–29 years of age with the APC of − 7.3% yearly (95%CI: − 12.5 to − 1.8), (Fig. 1, Table 3).

Results of Joinpoint regression analysis of T1DM incidence rates are presented in Table 3.

The significant higher incidence rate of T1DM was determined in the age group 5–9 years of age compared to the age groups 0–4, 15–19, 20–24 and 25–29 (Table 4).

There was a significantly higher incidence rate of T1DM in the age group 10–14 compared with the age groups 0–4, 15–19, 20–24 and 25–29 (Table 4).

Incidence rate of T2DM in the age group 25–29 was higher than of T1DM incidence rate (11.75 vs 8.22, *p* = 0.054) but the difference was not significant (Table 5).

## Discussion

The incidence of T1DM has increased worldwide over the past three decades. T1DM is predominantly a disease of children aged 0–14. Incidence of T1DM in European countries is between 5 and 10/100.000 per year and the countries of Eastern and Central Europe show lower incidence and an increasing trend in recent decades [1, 15, 19, 24].

We found that the incidence rates of T1DM were significantly higher in children from the age groups 5–9 and 10–14 in comparison to those aged 15–19 and young adults aged 20–29. The highest incidence rate of T1DM was recorded in the age group 10–14 and the lowest in adults aged 25–29.

According to the results obtained from the study on the prevalence of obesity in preschool children and primary school children in Serbia [25], the lowest obesity rates were reported in 7-year-old boys (6.2%), while the highest obesity prevalence rates were observed in 6-year-old boys (9.7%). The overall prevalence of overweight children (23.1%, including obesity) and obesity (6.9%) in Serbian primary school children points to the existence of obesity epidemic in Serbian children.

Literature data show similar results [14–16]. The incidence of T1DM increases with age in most populations with the highest incidence recorded in children aged 10–14 [9]. In the period 1982–2005 in Belgrade, Serbia, children aged 10–14 had the highest incidence rate of T1DM [12]. Similar data were found in the United States of America (USA) where incidence rate was the highest in children aged 10–14 and the ASR was 45.5/100.000 [2] compared with ASR of 21.10/100.000 in Serbian children of the same age.

The significantly increased incidence was observed in children in the age groups 5–9 and 10–14 years of age compared to the other age groups. Contrary to that, the lowest increase of incidence in the Republic of Srpska and Slovenia was noticed in the age group 10–14 years of age [21]. The highest increase in incidence of 5.7% yearly was observed in children aged 5–9. The highest average percentage yearly was recorded in boys and girls aged 5–9 in Belgrade during the period 1982 to 2005 [12].

The highest increase in the incidence in T1DM in both the Republic of Srpska (7.7% yearly) and Slovenia (4.3%) was observed in the youngest age group (0–4) and according to our findings, the increase of incidence in the age group 0–4 was 3.1% yearly and it was not significant. In this age group the incidence was the lowest in Serbia according to our findings. The lowest incidence rate in children aged 0–4 for both sexes were found in the study of Belgrade children from 1982 to 2005 [12].

Increasing trends in T1DM have been observed in the countries in our neighborhood such as in former Yugoslavian Republics of Slovenia, Montenegro, and Croatia [21]. The former Yugoslav republic, North Macedonia, is known as the “cold spot” of T1DM because of the lowest incidence of this DM type in the whole Europe [26].

After the age of 15 the incidence rate of T1DM significantly decreased and in the adults aged 25–29 the incidence of T2DM was higher than T1DM in adults from the same age group but the difference was not significant. In countries of the European Region overweight (OW) prevalence varies from 32 to 79% among men, and from 28 to 78% among women. The prevalence of obesity ranges from 5 to 23% among men and from 7 to 36% among women.

Increased incidence of T2DM in young adults can be a consequence of increased prevalence of obesity in Serbia, which is the main risk factor for the development of T2DM.

We found a decrease of T1DM incidence in Serbia in adolescents aged 15–19 and youths aged 20–24 and 25–29 years of age. In the age group 15–19 in Serbia, the incidence was 10.4/100000, and that is about ten times higher compared to the lowest registered incidence in the world on Mauritius (1.1/100,000) and nearly four times lower than the highest incidence which is recorded in Estonia (39.9/100,000) [20].

The highest incidence in Serbia was in the age group 10–14 (21.10/100,000) and it was 2.6 times higher than in the lowest recorded incidence in adults aged 25–29 (8.22/100,00).

Increasing trends of T1DM incidence were similar in children and adults (3% per year) in Italy and incidence rates were stable in the group 15–29 years of age (in the period 1991–1999) in the United Kingdom (UK) and in the group 15–39 years of age (period 1992–1996) in Finland [1].

Our results showed that there is no sufficient data concerning the T1DM incidence in young adults compared to data concerning children in Serbia, which is similar to the results of other studies [16, 18, 19]. This might be a consequence of small detection rate of specific autoantibodies and dosage of C-peptide as diagnostic criteria of T1DM in adults in Serbia and higher prevalence of T2DM risk factors in young adults especially in those above 25 years of age.

We found that the incidence of T2DM in the age group 25–29 was 1.4 times higher than the one for T1DM. This finding is similar to the findings from other studies [16, 23, 25, 27].

The results of our study showed that in Serbia after the age of 15 the incidence rate of T1DM is significantly decreasing. The risk of T1DM declines steeply after the age of 15 in European countries with high incidence rates, such as Finland [27] as well as in countries with low incidence, such as Lithuania [28] and Italy [26]. According to the results of the SEARCH study from the USA incidence rates in adults 15–29 years of age was 13.4/100,000 in the period 2002–2009 which was twofold lower than in children aged 0–14 [4, 29]. The incidence of T1DM in adults from Sweden had a very low completeness of ascertainment and a recent study has shown that the real incidence in adults up to 34 years of age was two to three times higher than previously reported [18].

So far, only two studies described a different clinical presentation of T1DM in young adults in comparison to children [18, 26]. A better quality as well as the amount of information about T1DM in adulthood will contribute to a better understanding of epidemiological characteristics of this type of DM, but it will also contribute to a faster implementation of the optimal therapy, the insulin therapy introduction and the prevention of the early onset of cardiovascular complications and premature death of the affected individuals [30].

The recognized misclassification of T1DM as T2DM is the main problem affecting studies on the epidemiology of T1DM in young adults [25]. According to the data of World Health Organization (WHO) the cause of T1DM is not known and it is not a preventable disease with current medical knowledge [6, 30].

## Conclusion

The increase of incidence in children aged 0–14 and the decrease after the 15 years of age showed that T1DM is predominantly a metabolic disease of children in Serbia. A significant increasing incidence trend was recorded in two age groups 5–9 and 10–14 years of age. The highest increase of incidence trend was in children aged 5–9 and the highest incidence rate was in children aged 10–14. A significant increase of T2DM incidence was observed in young adults aged 25–29. The increase in incidence rates in children, but not in young adults, suggests that the precipitating factors of children-onset disease may differ from those of adult-onset T1DM.

## Data Availability

These datasets are available in the [Public Health Institute of Serbia], link: [http://www.batut.org.rs/index.php?content=187]. Identifying/confident patient data will not be shared.

## Abbreviations

APC: Annual Percentage Change
ASR: Age-Standardized Rate
CI: Confidence Intervals
T1DM: Type 1 Diabetes Mellitus
T2DM: Type 2 Diabetes Mellitus
WHO: World Health Organization

## References

[1] World Health Organization. Global report on diabetes (2016). Available at: https://www.who.int/diabetes/global-report/.

[2] Atkinson AM, Eisenbarth SG, Aaron W, Michels WA (2014). Type 1 diabetes. Lancet.

[3] Bruno G, Gruden G, Songini M. Incidence of type 1 diabetes in age groups above 15 years: facts, hypothesis and prospects for future epidemiologic research. Acta Diabetol. 2016;53(3):339-47. 10.1007/s00592-015-0835-8. 10.1007/s00592-015-0835-8 26787492

[4] Rogers MAM, Kim C, Banerjee T (2017). Fluctuations in the incidence of type 1 diabetes in the United States from 2001 to 2015: a longitudinal study. BMC Med.

[5] Shaltout AA, Wake D, Thanaraj TA, Omar DM, Al-Abdul Razzaq D, Channanath A (2017). Incidence of type 1 diabetes has doubled in Kuwaiti children 0-14 years over the last 20 years. Pediatr Diabetes.

[6] DIAMOND Project Group (2006). Incidence and trends of childhood type 1 diabetes worldwide 1990–1999. Diabet Med.

[7] Incidence and Mortality of Diabetes Mellitus. Institute of Public Health of Serbia “Dr Milan Jovanović Batut”. Serbian Diabetes Registry. 2018: Report No 12.

[8] Todd JA (2010). Etiology of type 1 diabetes. Immunity..

[9] Maahs MD, West AN, Lawrence MJ, Mayer-Davis JE (2010). Epidemiology of type 1 diabetes. Endocrinol Metab Clin N Am.

[10] Pugliese A. The multiple origins of type 1 diabetes 2013. Available at: 10.1111/dme.12081.

[11] Forouhi GN, Nicholas J, Wareham JN. Epidemiology of diabetes. Medicine (Abingdon). 2014;42(12):698–702. 10.1016/j.mpmed.2014.09.007 PMC4282306 25568613

[12] Sipetic S, Maksimovic J, Vlajinac H, Ratkov I, Sajic S, Zdravković D (2005). Rising incidence of type 1 diabetes in Belgrade children aged 0-14 years in the period from 1982 to. J.Endocrinol.Invest.

[13] Bruno G, Cerutti F, Merletti F, Cavallo-Perin P, Gandolfo E, De Salvia A (2005). Residual b-cell function and male/female ratio are higher in incident young adults than in children: the registry of type 1 diabetes of the province of Turin, Italy, 1984–2000. Diabetes Care.

[14] Xia Y, Xie Z, Huang G, Zhou Z (2019). Incidence and trend of type 1 diabetes and the underlying environmental determinants. Diabetes Metab Res Rev.

[15] Diaz-Valencia PA, Bougneres P, Valleron AJ (2015). Global epidemiology of type 1 diabetes in young adults and adults: a systematic review. BMC Public Health.

[16] BerhanY, Waernbaum I, Lind T, Mollsten A, Dahlquist G (2011). For the Swedish childhood diabetes study group*. Thirty years of prospective nationwide incidence of childhood type 1 diabetes: the accelerating increase by time tends to level off in Sweden. Diabetes.

[17] Diaz-Valencia PA, Bougneres P, Valleron AJ. Covariation of the incidence of type 1 diabetes with country characteristics available in public databases. PloS One. 2015; Available at: 10.1371/journal.pone.0118298. 10.1371/journal.pone.0118298 PMC4338253 25706995

[18] Borg H, Arnqvist HJ, Bjork E, Bolinder J, Eriksson JW, Nyström L (2003). Evaluation of the new ADA and WHO criteria for classification of diabetes mellitus in young adult people (15–34 yrs) in the diabetes incidence study in Sweden (DISS). Diabetologia.

[19] Patterson C, Guariguata L, Dahlquist G, Soltész G, Graham Ogle G, Silink M (2013). Diabetes in the young-a global view and worldwide estimates of numbers of children with type 1 diabetes. Diabetes Res Clin Pract.

[20] Leslie RD, Delli CM (2004). Age-dependent influences on the origins of autoimmune diabetes: evidence and implications. Diabetes.

[21] Rawshani A, Sattar N, Franzén S, Rawshani A, Hattersley AT (2018). Excess mortality and cardiovascular disease in young adults with type 1 diabetes in relation to age at onset: a nationwide, register-based cohort study. Lancet.

[22] Segi M (1960). Cancer mortality for selected sites in 24 countries (1950–57).

[23] The Joinpoint Regression Program version 4.6.0.0. Available at: http: //surveillance.cancer.gov/joinpoint.

[24] Craig ME, Hattersley A, Donaghue KC. Definition, epidemiology and classification of diabetes in children and adolescents. Pediatr Diabetes. 2009; 10(Suppl. 12):3–12. 10.1111/j.1399-5448.2009.00568.x 19754613

[25] Djordjic V, Radisavljevic S, Milanovic I, Bozic P, Grbic M, Jorga J (2016). WHO European childhood obesity surveillance initiative in Serbia: a prevalence of overweight and obesity among 6-9-year-old school children. J Pediatr Endocrinol Metab.

[26] Radosevic B, Bukara-Radujkovic G, Miljkovic V, Pejicic S, Bratina N, Battelino T (2013). The incidence of type 1 diabetes in republic of Srpska (Bosnia and Herzegovina) and Slovenia in the period 1998–2010. Pediatr Diabetes.

[27] Lammi N, Blomstedt PA, Moltchanova E, Tuomilehto J, Karvonen M (2008). Marked temporal increase in the incidence of type 1 and type 2 diabetes among young adults in Finland. Diabetologia.

[28] Patterson CC, Gyurus E, Rosenbauer J, Cinek O, Neu A, EURODIAB Study Group (2012). Trends in childhood type 1 diabetes incidence in Europe during 1989–2008: evidence of non-uniformity over time in rates of increase. Diabetologia.

[29] Ostrauskas R, Žalinkevičius R, Jurgevičienė N, Radzevičienė L, Lina LL (2011). The incidence of type 1 diabetes mellitus among 15–34 years aged Lithuanian population: 18-year incidence study based on prospective databases. BMC Public Health.

[30] Bruno G, Novelli G, Panero F, Perotto M, Monasterolo F, Bono G (2009). The incidence of type 1 diabetes in increasing in both children and young adults in northern Italy: 1984-2004 temporal trends. Diabetologia..

